# Does muscle strength predict working memory? A cross-sectional fNIRS study in older adults

**DOI:** 10.3389/fnagi.2023.1243283

**Published:** 2023-10-09

**Authors:** Zhidong Cai, Xing Wang, Qiang Wang

**Affiliations:** ^1^Department of Physical Education, Suzhou University of Science and Technology, Suzhou, China; ^2^School of Physical Education, Shanghai University of Sport, Shanghai, China; ^3^School of Physical Education, Guangzhou Sport University, Guangzhou, China

**Keywords:** cognition, muscle strength, 30-s sit-up, grip strength, N-back, functional near infrared spectroscopy

## Abstract

**Objective:**

Previous research has primarily focused on the association between muscle strength and global cognitive function in older adults, while the connection between muscle strength and advanced cognitive function such as inhibition and working memory (WM) remains unclear. This study aimed to investigate the relationship among muscle strength, WM, and task-related cortex hemodynamics.

**Methods:**

We recruited eighty-one older adults. Muscle strength was measured using a grip and lower limb strength protocol. We measured the WM performance by using reaction time (RT) and accuracy (ACC) in the N-back task and the cortical hemodynamics of the prefrontal cortex (PFC) by functional near-infrared spectroscopy (fNIRS).

**Results:**

We found positive correlations between grip strength (*p* < 0.05), 30-s sit-up (*p* < 0.05) and ACC, negative correlation between grip strength (*p* < 0.05) and RT. Furthermore, we observed positive correlations between grip strength and the level of oxygenated hemoglobin (HbO_2_) in dorsolateral prefrontal cortex, frontopolar area, ventrolateral prefrontal cortex (*p* < 0.05), and negative correlations between grip strength and the level of deoxygenated hemoglobin (Hb) in left dorsolateral prefrontal cortex, frontopolar area, left ventrolateral prefrontal cortex (*p* < 0.05). Additionally, we noticed positive correlations between RT and the level of Hb in left dorsolateral prefrontal cortex, right frontopolar area (*p* < 0.05), and negative correlations between RT and the level of HbO_2_ in left dorsolateral prefrontal cortex, frontopolar area (*p* < 0.05). However, the cortical hemodynamics did not mediate the relationship between muscle strength and WM performance (RT, ACC).

**Conclusion:**

The grip strength of older adults predicted WM in the cross-section study. The level of hemodynamics in PFC can serve as a predictor of WM.

## Introduction

Working memory (WM) was considered to be an advanced cognitive function that involves the simultaneous storage and processing of information. Research showed that WM declines with age (Bopp and Verhaeghen, 2020), with a marked decrease after the age of 60 (Elliott et al., 2011). WM was closely related to prefrontal cortex (PFC) activation, and changes in PFC activation indicated aging of WM (Yaple et al., 2019). Studies found that the prefrontal cortex (PFC) was activated during WM tasks (Kobori et al., 2015), and the dorsolateral prefrontal cortex (DLPFC) was continuously activated during delayed response tasks. Studies showed that older adults experienced increased PFC activation (Agbangla et al., 2019), and more bilateral PFC activation, during N-back tasks (Vermeij et al., 2012). One study found that older adults had greater right DLPFC activation at low WM load, but less right DLPFC activation at high WM load compared to younger adults (Wijeakumar et al., 2017). Taken together, increased activation of the PFC was related to improved WM performance, but the activation of the PFC decreased when the WM load exceeded the cognitive ability of the older adults.

Recent reviews found that preserving muscle strength was beneficial for brain health and cognitive function (Herold et al., 2019; Landrigan et al., 2020). In this context, a study showed that decreased muscle strength (grip strength, 5 sit-ups) in older adults was found to be associated with worse WM performance (digit span test) and accompanied by medial temporal cortex atrophy (Liu et al., 2022). Upper and lower extremity strength was an important marker of brain health and might share a common neural basis with higher cognitive function (Fritz et al., 2017; Carson, 2018; Shaughnessy et al., 2020). The “muscle-brain axis” hypothesis suggested that changes in muscle mass and/or muscle strength were involved in the development of cognitive decline through changes in brain structure and function (Burtscher et al., 2021). A study explored the mediation effect of cortical hemodynamics between grip strength and WM in young adults, but unfortunately, it was not found and recommended to be examined in older adults (Herold et al., 2021). Another study found a mediation effect of cortex hemodynamics between cardiorespiratory fitness and cognitive function in older adults (Hyodo et al., 2016). Given that cardiorespiratory fitness and muscle strength are both important components of health and fitness, so cortical hemodynamics may also mediate the relationship between muscle strength and WM.

Previous research primarily focused on the association between muscle strength and global cognitive function in older adults (Soga et al., 2018; Marston et al., 2019; Landrigan et al., 2020), while the connection between muscle strength and advanced cognitive function such as inhibition and WM remains unclear. Additionally, there was a lack of understanding regarding the neurophysiological mechanisms that underlie the relationship between higher levels of muscle strength and better WM performance. Therefore, this study aimed to investigate the interplay among muscle strength, WM, and prefrontal cortex hemodynamics (specifically, increased concentration of oxyhemoglobin and decreased concentration of deoxygenated hemoglobin) in older adults. We hypothesized that muscle strength (e.g., grip strength, 30-s sit-up) would be positively correlated with (1) WM, (2) prefrontal activation, and that WM would positively correlate with prefrontal activation. Furthermore, we hypothesized that changes in cortical hemodynamics would mediate the relationship between muscle strength and WM.

## Methods

### Participants and study design

In this study, we recruited older adults from three nursing homes located in the Songjiang, Qingpu, and Hongkou districts of Shanghai, China. There was no statistical difference in age, education level, cognitive status among the older adults in the three recruitment sites. The eligibility criteria for participation were as follows: participants had to be over 75 years of age, right-handed, without exercise contraindications according to the American College of Sports Medicine healthy fitness pre-exercise screening questionnaire and had normal vision and hearing. Exclusion criteria included self-reported history of cardiac surgery, asthma, severe diabetes, severe hypertension, severe mental illness, dementia or the use of anti-cognitive drugs, severe motor system disease, history of neurological diseases, and recent use of elastic bands for exercise. This study was conducted following the latest version of the Declaration of Helsinki and was approved by the Ethics Committee of Shanghai University of Sport (No.102772020RT060).

### Experimental procedures

All participants were required to attend the laboratory on two occasions, with a minimum interval of 3 days between visits. During the first visit, participants received a brief introduction to the study, provided their consent, and completed a series of questionnaires assessing various factors such as demographics, muscle strength (measured by a handhold dynamometer and 30-s sit-up test), handedness (determined by the Edinburgh Handedness Inventory), and cognitive state (assessed by the Montreal Cognitive Assessment). During the second visit, fNIRS was utilized to record cortical hemodynamics while completing the N-back task.

### Muscle strength

We chose grip strength and 30-s sit-up to measure the muscle strength of older adults. Grip strength was measured using a handhold dynamometer (Camry EH101, Senssun, China). Participants stood upright with their arms straight at their sides and their wrists in a neutral position. They were instructed to squeeze the handhold dynamometer as hard as they could for 3 s. Every participant conducted three trials for each hand and changed after one trial. The interval between each trial was 15 s, which was enough time to recover, and ensure the consistency of the test. The maximal grip strength (MGS) of the three trials for each hand was used for further analysis. To account for the influence of body composition, MGS was normalized to the participant’s body mass index (BMI) using the following equation: normalized grip strength (NGS) = MGS (kg) /BMI (kg/m^2^) (Mcgrath et al., 2020).

The 30-s sit-up test (30SUP) measured lower limb strength. 30SUP had good reliability and validity for evaluating the lower limb muscle strength of older adults, and an evaluation model had been established for this test (Bai et al., 2020). During the test, participants stood in front of a chair that was approximately 43 cm high and crossed their arms in front of their chest. They were then required to stand up and sit down repeatedly as quickly as possible for 30 s. Participants must maintain a straight back and cannot touch the back of the chair. The number of times they stood up in 30 s was recorded. To account for body composition, 30SUP and normalized 30 s sit-up (30NSUP) were used for analysis, 30NSUP = 30SUP (times) /BMI (kg/m^2^) (Tan et al., 2012).

### N-back task paradigm

We assessed WM using a computerized version of the Arabic numerals N-back task. The N-back task was chosen due to its previous usage in examining prefrontal cortex activation with fNIRS in literature (Stute et al., 2020). The N-back task consisted of three blocks (i.e., 0-back, 1-back, 2-back), which were repeated 5 times. Stimuli were black Arabic numerals (0 ~ 9) with a stimulus object size of 3 cm × 3 cm, which were presented in the center of a screen on a grey background. Each stimulus was presented for 500 ms followed by a response interval of 2000 ms. We used E-prime (version 2.0, Psychology Software Tools, Sharpsburg, PA, USA) for stimulus presentation, reaction time (RT), and accuracy (ACC) recordings.

In the 0-back condition, the Arabic numeral “0” served as the target, whereas in the 1-back and 2-back conditions, numerals from 0 to 9 were randomly presented. Before the experiment started, the participants sat comfortably in a quiet, dim room, viewing a screen about 70 cm distance. Participants were required to determine whether each displayed numeral matched the numeral in the previous trial (1-back condition) or two trials before (2-back condition). If the stimulus was a target, participants used their right index finger to press “1,” and if it was a non-target, used their right middle finger to press “2” on the mini keyboard. Participants were instructed to respond as quickly and as accurately as possible. The total duration of the experiment was about 15 min. RT and ACC of the N-back task were used as outcome measures for WM performance. Responses with an RT of less than 200 ms or greater than 2,500 ms were excluded.

### fNIRS measurement

**Figure 1:**
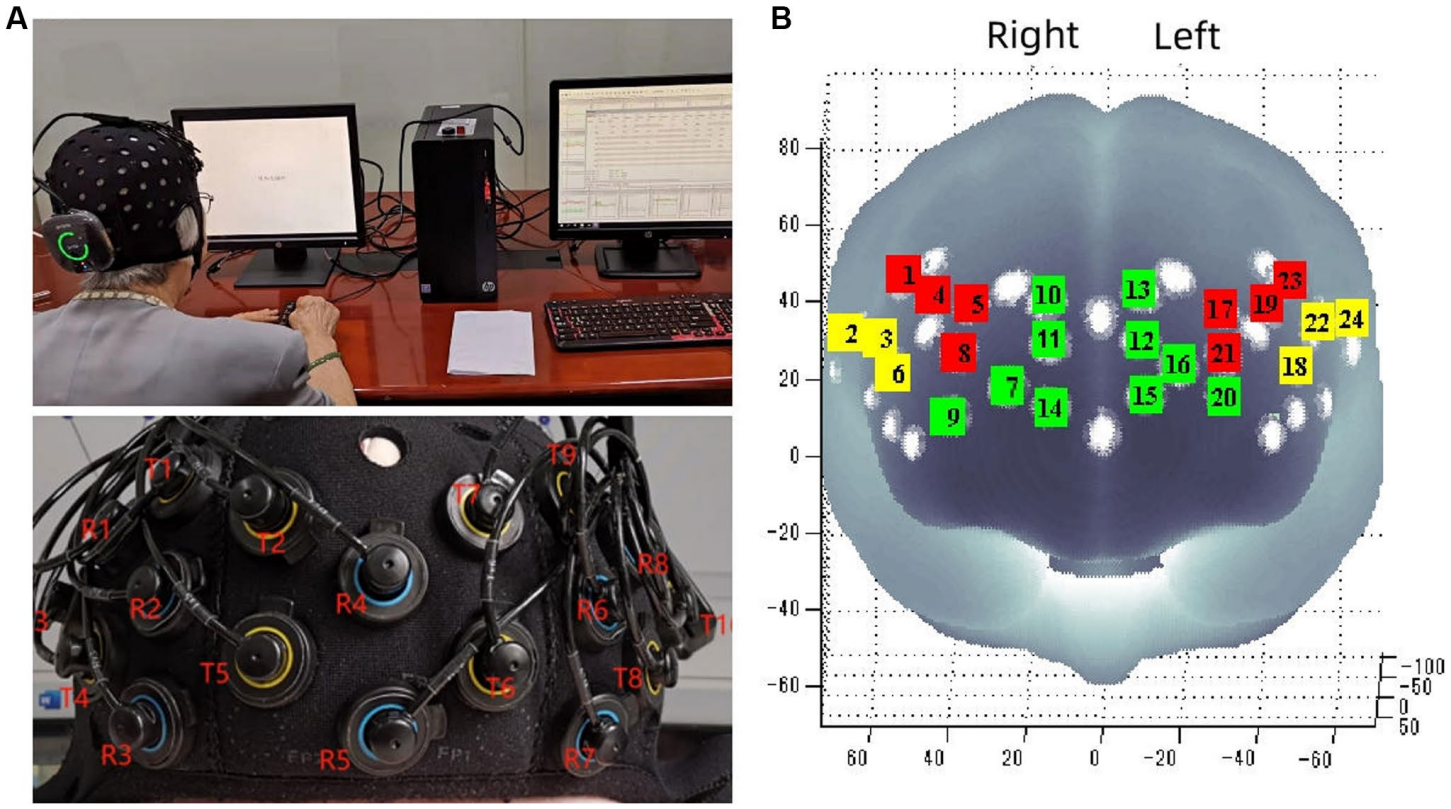
Portable near infrared brain imaging system Brite24 and ROI Setup. **(A)** Portable near infrared brain imaging system Brite24 and the distribution of light emitters and receptors. **(B)** Region of interest, T, light emitters; R, light receptors; red square, DLPFC; yellow square, VLPFC; green square, PFA.

We recorded changes in HbO_2_ and Hb concentrations by using a portable 24-channel continuous-wave fNIRS system (Brite24, Artinis Medical Systems, Netherlands; Figure 1A). The baseline was the blood oxygen concentration 30 s before the N-back task. The fNIRS system consists of 10 light emitters (emitting light at wavelengths of 760 and 850 nm), and 8 light receptors. The distance between each emitter and receptor was 3 cm. fNIRS optodes were located according to the 10–20 EEG system (Jurcak et al., 2007). We performed a virtual and probabilistic spatial registration using the software fNIRS Optodes’ Location Decider (Zimeo et al., 2018) and the Broadman atlas (Rorden and Brett, 2000). The region of interest (ROI) (Figure 1B) we measured included the right dorsolateral prefrontal cortex (R-DLPFC) (ch1, ch4, ch5, ch8), left dorsolateral prefrontal cortex (L-DLPFC) (ch17, ch19, ch21, ch23), left frontopolar area (L-FPA) (ch12, ch13, ch15, ch16, ch20), right frontopolar area (R-FPA) (ch7, ch9, ch10, ch11, ch14), right ventrolateral prefrontal cortex (R-VLPFC) (ch2, ch3, ch6), left ventrolateral prefrontal cortex (L-VLPFC) (ch18, ch22, ch24).

### Data analysis

#### fNIRS data processing

The fNIRS data were preprocessed using the software NIR-SPM (version 4 KAIST Seoul Korea). Firstly, we used principal component analysis to remove physiological noise from each channel and set the parameter to 0.97. Secondly, we used the Wavelet-minimum description length (Wavelet-MDL) method for high-pass filtering, mainly to remove noise (head motion, heartbeat, etc.) and baseline drift. Wavelet-MDL applied wavelet transform to near-infrared time series and decomposed it into different scales of deviation, hemodynamic signals, and noise components. We used the hemodynamic response function for low-pass filtering of fNIRS data. Then, the task effect was integrated by the general linear model, the task fitting reference wave was used to infer the parameter estimation, and the precoloring method was used to adjust the time autocorrelation of this process (Bai et al., 2016).

#### Statistical analysis

The statistical analysis was conducted using IBM SPSS (Version 22, Chicago, Illinois, USA). The normal distribution of the data was assessed using the Shapiro–Wilk test. As most of the fNIRS data was not normally distributed, a non-parametric partial correlation analysis was used. In the second step, we examined the bivariate relationships between muscle strength (grip strength, 30-s sit-up) and WM (RT, ACC), WM and cortical hemodynamics (HbO_2_, Hb), cortical hemodynamics and muscle strength by calculating non-parametric partial correlations while controlling for age and sex. In the third step, Smart PLS software (Version 3.0 GmbH, Germany) was used for the mediation analysis. A mediation analysis was performed to investigate whether cortical hemodynamics mediate the relationship between muscle strength and WM. The level of statistical significance was set to α = 0.05 in the analysis.

## Results

### Participants characteristics

A total of 92 older adults were recruited from the three nursing homes, with 85 participants completing all tests. Four participants were excluded due to missing functional near-infrared spectroscopy (fNIRS) data, resulting in a final sample size of 81 individuals. Participants’ characteristics can be found in Table 1.

**Table 1:** Basic information of the participants.

	Total (*n* = 81)	Female (*n* = 48)	Male (*n* = 33)
Age (year)	86.39 ± 9.00	85.64 ± 10.00	87.94 ± 8.00
Height (cm)	158.29 ± 7.50	155.46 ± 6.50	164.13 ± 11.30
Weight (kg)	55.30 ± 19.50	52.15 ± 13.00	61.78 ± 18.50
BMI (kg/m^2^)	21.97 ± 5.50	21.52 ± 4.70	22.88 ± 5.10
MoCA (point)	23.37 ± 7.50	22.39 ± 9.00	25.38 ± 3.80
Handedness	right	right	right
Education level (years)	9.35 ± 4.58	8.75 ± 4.71	10.21 ± 4.31

BMI, body mass index; MoCA, Montreal cognitive assessment; data are median ± interquartile range.

### Correlation between muscle strength and WM performance

#### Correlation between grip strength and WM performance

**Figure 2:**
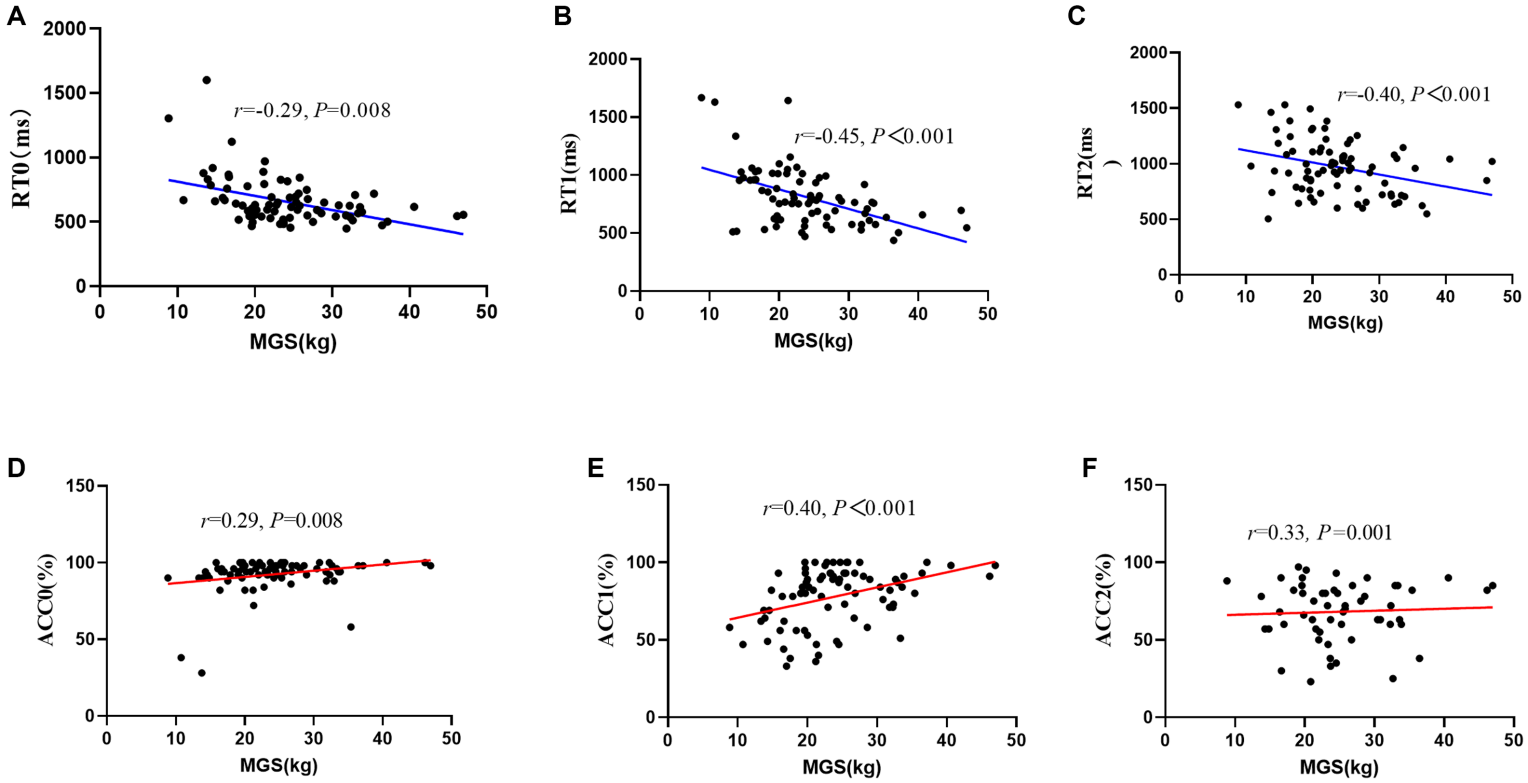
Scatter plot of the relationship between maximal grip strength and working memory performance (RT/ACC). **(A)** RT0, reaction time during 0-back; **(B)** RT1, reaction time during 1-back; **(C)** RT2, reaction time during 2-back; **(D)** ACC0, accuracy during 0-back; **(E)** ACC1, accuracy during 1-back; **(F)** ACC2, accuracy during 2-back; MGS, maximal grip strength.

After controlling for the variables of age and sex, there was a negative correlation between MGS/NGS and working memory, such that those with greater MGS/NGS displayed worse RT during all conditions (i.e., MGS: 0-back: *r* = −0.29, *p* = 0.008, 1-back: *r* = −0.45, *p* < 0.001, 2-back: *r* = −0.40, *p* < 0.001; NGS: 0-back: *r* = −0.24, *p* = 0.023, 1-back: *r* = −0.44, *p* < 0.001, 2-back: *r* = −0.37, *p* < 0.001), and with greater MGS/NGS displayed better ACC during all conditions (i.e., MGS: 0-back: *r* = 0.29, *p* = 0.008, 1-back: *r* = 0.40, *p* < 0.001, 2-back: *r* = 0.33, *p* = 0.001; NGS: 0-back: *r* = 0.28, *p* = 0.010; 1-back: *r* = 0.33, *p* = 0.002, 2-back: *r* = 0.32, *p* = 0.003; Figure 2).

#### Correlation between 30-s sit-up and WM performance

**Figure 3:**
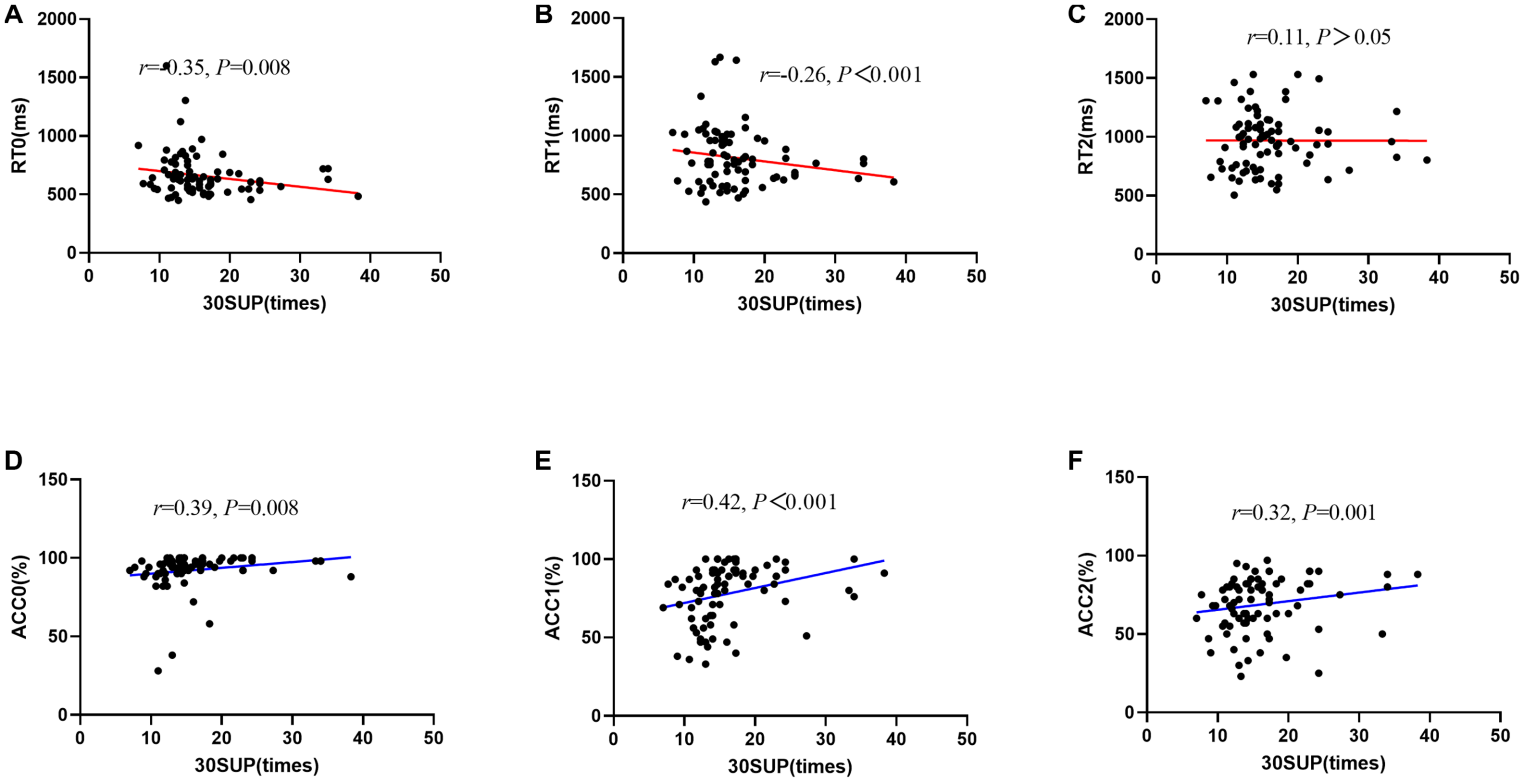
Scatter plot of the relationship between 30-s sit-up and working memory performance (RT/ACC). **(A)** RT0, reaction time during 0-back; **(B)** RT1, reaction time during 1-back; **(C)** RT2, reaction time during 2-back; **(D)** ACC0, accuracy during 0-back; **(E)** ACC1, accuracy during 1-back; **(F)** ACC2, accuracy during 2-back; MGS, maximal grip strength.

After controlling for the variables of age and sex, there was a negative correlation between 30SUP/30NSUP and working memory, such that those with greater 30SUP/30NSUP displayed worse RT during some conditions (i.e., 30SUP: 0-back: *r* = −0.35, *p* = 0.008, 1-back: *r* = −0.26, *p* < 0.001; 30NSUP: 0-back: *r* = −0.22, *p* = 0.023), and with greater 30SUP/30NSUP displayed better ACC during some conditions (30SUP: 0-back: *r* = 0.39, *p* = 0.008, 1-back: *r* = 0.42, *p* < 0.001, 2-back: *r* = 0.32, *p* = 0.001; 30NSUP: 1-back: *r* = 0.25, *p* = 0.002; Figure 3; Table 2).

**Table 2:** Correlation between Hb and muscle strength and N-back performance.

Ch	Hb	Muscle strength				RT	ACC	N-back
		MGS	NGS	30SUP	30NSUP			
1	−0.035 ± 0.672	0.075	0.049	−0.168	−0.270*	0.005	−0.096	0-back
17	0.096 ± 0.368	−0.129	−0.157	0.011	0.040	0.243*	−0.032	0-back
19	0.047 ± 0.214	−0.277*	−0.313*	−0.011	0.034	0.267*	−0.325*	0-back
22	0.084 ± 0.210	−0.112	−0.227*	−0.036	0	0.172	−0.129	0-back
23	0.034 ± 0.487	−0.222*	−0.114	−0.101	0.046	0.140	−0.287*	0-back
9	0.068 ± 0.261	−0.356*	−0.351*	0.067	−0.021	0.249*	−0.250*	1-back
13	0.042 ± 0.270	−0.233*	−0.138	−0.084	0.078	0.006	−0.146	1-back
19	0.051 ± 0.347	−0.356*	−0.351*	0.067	−0.021	0.326*	−0.271*	1-back
23	0.065 ± 0.295	−0.235*	−0.223*	−0.207	−0.292*	0.313*	0.069	1-back
5	−0.032 ± 0.403	0.009	0.050	−0.058	−0.010	0.052	−0.221*	2-back
10	−0.022 ± 0.347	−0.094	−0.093	−0.063	−0.068	0.018	−0.269*	2-back
13	0.006 ± 0.329	−0.070	−0.092	0.025	0.003	0.098	−0.259*	2-back
17	0.073 ± 0.391	0.010	−0.103	0.059	0.002	0.047	−0.265*	2-back
20	0.049 ± 0.310	−0.288*	−0.195	0.136	0.021	−0.186	0.102	2-back
22	0.065 ± 0.407	0.010	−0.160	−0.032	−0.074	0.053	−0.262*	2-back

Ch, channel; Hb, deoxygenated hemoglobin; MGS, maximal grip strength; NGS, normalized grip strength; 30SUP, 30-s sit-up; 30NSUP, 30-s normalized sit-up; RT, reaction time; ACC, accuracy, the covariates were age and sex, *p < 0.05.

### Correlation between muscle strength and cortical hemodynamics

#### Correlation between muscle strength and oxygenated hemoglobin

We observed a positive correlation between grip strength and the HbO_2_ level in L-DLPFC (MGS: channel 19: *r* = 0.212, *p* = 0.034), R-DLPFC (NGS: channel 8: *r* = 0.222, *p* = 0.033), R-FPA (NGS: channel 11: *r* = 0.206, *p* = 0.041) during 0-back; between MGS and the HbO_2_ level in VLPFC (channel 3: *r* = 0.282, *p* = 0.009, channel 18: *r* = 0.321, *p* = 0.003), DLPFC (channel 4: *r* = 0.309, *p* = 0.005, channel 17: *r* = 0.253, *p* = 0.018, channel 19: *r* = 0.299, *p* = 0.008), L-FPA (channel 13: *r* = 0.247, *p* = 0.021) during 1-back; between NGS and the HbO_2_ level in R-DLPFC (channel 4: *r* = 0.315, *p* = 0.003) during 2-back. Furthermore, 30NSUP showed a positive correlation with the HbO_2_ in R-DLPFC (channel 5: *r* = 0.230, *p* = 0.008) during the 2-back task.

#### Correlation between muscle strength and deoxygenated hemoglobin

We observed a negative correlation between grip strength and the Hb level in L-DLPFC (MGS: channel 19: *r* = −0.277, *p* = 0.011, channel 23: *r* = −0.222, *p* = 0.034; NGS: channel 19: *r* = −0.313, *p* = 0.003), L-VLPFC (NGS: channel 22: *r* = −0.227, *p* = 0.011) during 0-back; between grip strength and the Hb level in FPA (MGS: channel 9: *r* = −0.356, *p* < 0.001, channel 13: *r* = −0.233, *p* = 0.003; NGS: channel 9: *r* = −0.351, *p* < 0.001), L-DLPFC (MGS: channel 19: *r* = −0.356, *p* = 0.001, channel 23: *r* = −0.235, *p* = 0.006; NGS: channel 19: *r* = −0.35, *p* < 0.001, channel 23: *r* = −0.223, *p* = 0.028) during 1-back; between MGS and the Hb in L-FPA (channel 20: *r* = −0.288, *p* = 0.014) during the 2-back task. Furthermore, we observed a significant negative correlation between 30NSUP and the Hb level in DLPFC (channel 1: 0-back: *r* = −0.270, *p* = 0.016, channel 23: 1-back: *r* = −0.292, *p* = 0.008).

### Correlation between WM performance and cortical hemodynamics

#### Correlation between WM performance and oxygenated hemoglobin

During the 0-back task, a significant negative correlation was found between RT and the HbO_2_ in L-DLPFC (channel 17: *r* = −0.240, *p* = 0.028, channel 19: *r* = −0.288, *p* = 0.003, channel 23: *r* = −0.250, *p* = 0.032), while a positive correlation was observed between ACC and the HbO_2_ in FPA (channel 11: *r* = 0.246, *p* = 0.019, channel 15: *r* = 0.267, *p* = 0.005), L-DLPFC (channel 23: *r* = 0.299, *p* = 0.001). During the 1-back task, a negative correlation was found between RT and the HbO_2_ in FPA (channel 11: *r* = −0.256, *p* = 0.025, channel 12: *r* = −0.253, *p* = 0.027, channel 16: *r* = −0.223, *p* = 0.028), L-DLPFC (channel 23: *r* = −0.237, *p* = 0.002). During the 2-back task, a positive correlation was found between ACC and the HbO_2_ in L-DLPFC (channel 23: *r* = 0.263, *p* = 0.011; Table 3).

**Table 3:** Correlation between HbO_2_ and muscle strength and N-back performance.

Ch	HbO_2_	Muscle strength				RT	ACC	N-back
		MGS	NGS	30SUP	30NSUP			
8	0.097 ± 0.325	0.152	0.222*	0.024	0.009	−0.081	0.049	0-back
11	0.071 ± 0.622	0.094	0.206*	0.079	0.056	−0.009	0.246*	0-back
14	0.130 ± 0.407	−0.112	−0.123	0.035	0.080	−0.073	0.267*	0-back
15	0.089 ± 0.464	−0.132	−0.120	−0.001	−0.005	−0.030	0.299*	0-back
17	0.065 ± 0.492	0.157	0.027	0.020	−0.049	−0.240*	−0.071	0-back
19	0.052 ± 0.643	0.212*	0.082	0.013	0.034	−0.288*	−0.076	0-back
23	0.138 ± 0.586	0.051	0.056	−0.041	−0.062	−0.250*	0.305*	0-back
3	0.181 ± 0.581	0.282*	0.117	0.087	0.019	0.161	0.220	1-back
4	0.132 ± 0.633	0.309*	0.056	0.008	0.016	0.001	−0.049	1-back
11	0.040 ± 0.513	−0.229	0.001	0.024	0.159	−0.256*	−0.160	1-back
12	0.125 ± 0.569	−0.175	0.036	−0.106	−0.115	−0.253*	−0.102	1-back
13	0.100 ± 0.491	0.247*	0.041	0.035	0.119	0.033	−0.005	1-back
16	0.090 ± 0.435	−0.109	−0.019	−0.124	−0.108	−0.223*	0.026	1-back
17	0.126 ± 0.661	0.253*	0.083	0.100	0.022	0.072	0.143	1-back
18	0.073 ± 0.483	0.321*	0.183	−0.138	−0.048	0.043	0.099	1-back
19	0.087 ± 0.551	0.299*	−0.035	−0.086	−0.128	−0.170	0.150	1-back
23	0.118 ± 0.597	0.104	0.004	0.086	0.010	−0.237*	0.022	1-back
4	0.155 ± 0.604	0.139	0.315*	−0.016	0.060	0.143	0.174	2-back
5	0.229 ± 0.838	0.125	0.062	0.088	0.230*	0.082	0.157	2-back
10	0.166 ± 0.832	−0.031	−0.139	0.008	0.062	−0.240*	0.125	2-back
23	0.138 ± 0.586	0.008	−0.053	0.057	0.045	0.115	0.263*	2-back
22	0.065 ± 0.407	0.010	−0.160	−0.032	−0.074	0.053	−0.262*	2-back

Ch, channel; HbO_2_, oxygenated hemoglobin; MGS, maximal grip strength; NGS, normalized grip strength; 30SUP, 30-s sit-up; 30NSUP, 30-s normalized sit-up; RT, reaction time; ACC, accuracy, the covariates were age and sex, *p < 0.05.

#### Correlation between WM performance and deoxygenated hemoglobin

During the 0-back task, a significant positive correlation was found between RT and the Hb in L-DLPFC (channel 17: *r* = 0.243, *p* = 0.026, channel 19: *r* = 0.267, *p* = 0.005), while a negative correlation was observed between ACC and the Hb in L-DLPFC (channel 19: *r* = −0.325, *p* = 0.001, channel 23: *r* = −0.287, *p* = 0.003). Furthermore, a significant positive correlation was observed between 1-back RT and the Hb in R-FPA (channel 9: *r* = −0.249, *p* = 0.021), L-DLPFC (channel 19: *r* = 0.326, *p* = 0.001, channel 23: *r* = 0.313, *p* = 0.001), and a significant negative correlation was found between ACC of 0-back and the Hb in L-DLPFC (channel 19: *r* = −0.325, *p* = 0.001, channel 23: *r* = −0.287, *p* = 0.003). Lastly, there was a significant negative correlation between 2-back ACC and the Hb in DLPFC (channel 5: *r* = −0.221, *p* = 0.037, channel 17: *r* = −0.265, *p* = 0.005), FPA (channel 10: *r* = −0.269, *p* = 0.005, channel 13: *r* = −0.259, *p* = 0.016), L-VLPFC (channel 22: *r* = −0.262, *p* = 0.014).

### Mediation analysis

**Figure 4:**
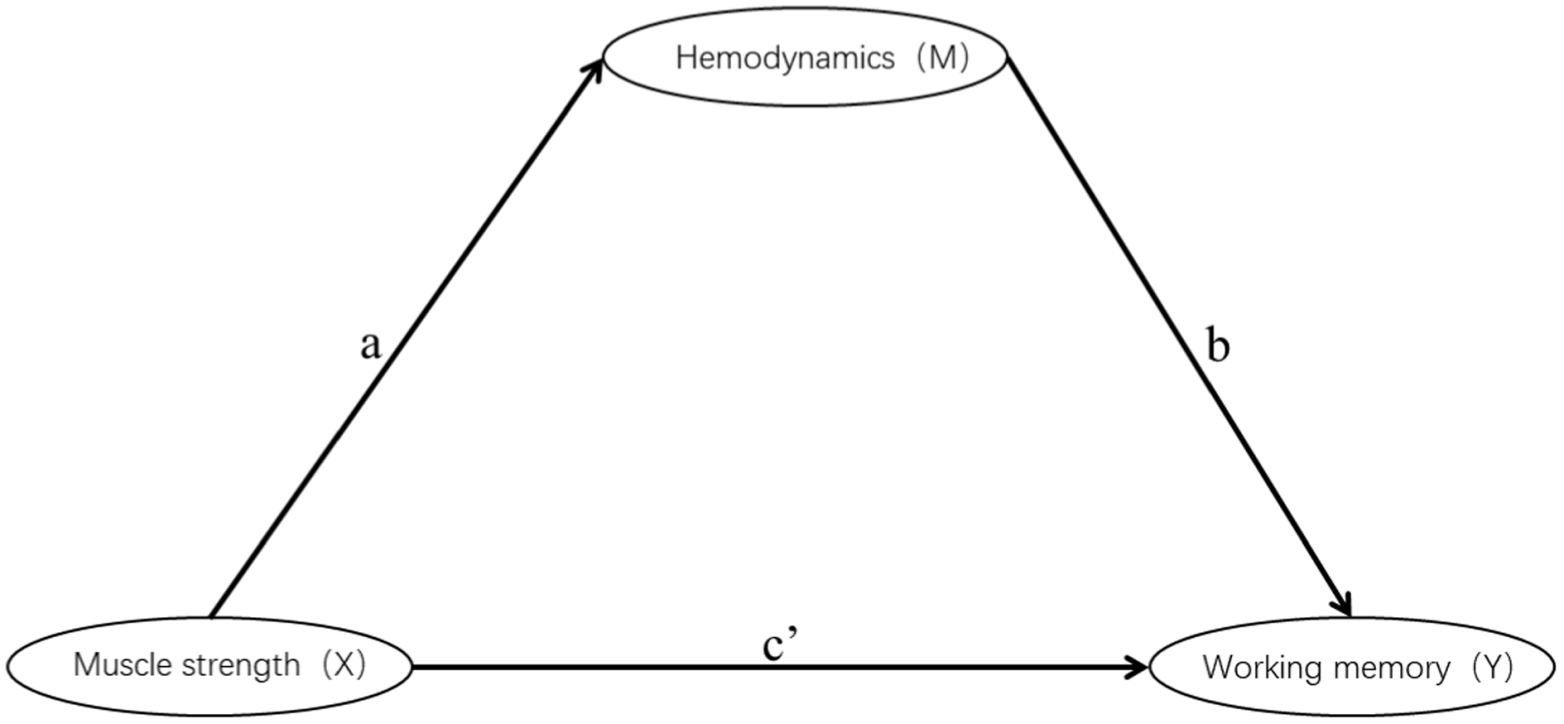
Schematic diagram of mediation.

This study only conducted mediation analysis if the path model satisfied two conditions: firstly, non-parametric partial correlation analysis indicated a significant correlation between the mediator and the independent or dependent variable; secondly, the correlation coefficient between the mediator and the dependent or independent variable was at least 0.20 (Ferguson, 2009; Figure 4). Based on these conditions, 13 channels (27 path models) for HbO_2_ and 11 channels (23 path models) for Hb satisfied the requirements for mediation analysis (Tables 4, 5).

**Table 4:** Mediation model test of Hb.

Path mode	Path A			Path B			Path C′		
	Coefficient	*t*	*p*	Coefficient	*t*	*p*	Coefficient	*t*	*p*
S-8-RT0	−0.253	2.756	0.006	0.117	0.144	0.480	−0.431	5.376	<0.001
S-8-ACC0	−0.255	2.727	0.006	−0.046	0.421	0.674	0.262	2.221	0.026
S-14-RT0	−0.239	2.165	0.031	0.268	1.318	0.188	−0.401	5.670	<0.001
S-14-ACC0	−0.240	2.145	0.032	0.119	1.559	0.119	0.305	2.233	0.026
S-20-ACC0	0.021	0.032	0.749	0.258	2.506	0.012	0.254	2.224	0.026
S-20-RT0	0.231	2.092	0.037	−0.231	1.272	0.203	−0.412	6.362	<0.001
S-22-ACC0	−0.203	1.627	0.104	0.005	0.068	0.946	0.198	2.316	0.021
S-22-RT0	−0.195	1.506	0.132	0.201	1.475	0.140	−0.381	5.732	<0.001
S-1-RT0	−0.096	0.922	0.357	−0.094	1.187	0.235	−0.428	7.131	<0.001
S-1-ACC0	−0.102	0.825	0.410	0.043	0.448	0.654	0.264	2.505	0.012
S-23-ACC0	−0.021	0.204	0.839	−0.024	0.880	0.556	0.246	2.077	0.038
S-23-RT0	−0.022	0.198	0.843	0.022	0.150	0.881	−0.420	6.939	<0.001
S-9-RT1	0.055	0.651	0.515	−0.323	2.387	0.017	−0.479	5.981	<0.001
S-9-ACC1	0.049	0.654	0.513	0.227	3.271	0.001	0.458	7.204	<0.001
S-20-RT1	0.264	3.245	0.001	−0.189	1.422	0.155	−0.417	5.186	<0.001
S-20-ACC1	0.242	2.567	0.010	0.166	1.538	0.124	0.407	6.158	<0.001
S-1-RT1	0.013	0.084	0.933	−0.169	1.669	0.095	−0.463	4.852	<0.001
S-1-ACC1	−0.087	0.665	0.060	−0.050	0.424	0.671	0.444	5.682	<0.001
S-23-RT1	−0.036	0.299	0.765	−0.084	0.626	0.531	−0.467	6.434	<0.001
S-23-ACC1	−0.029	0.266	0.790	0.110	1.097	0.273	0.453	6.733	<0.001
S-20-ACC2	0.098	0.644	0.520	0.132	1.723	0.085	0.385	4.126	<0.001
S-22-ACC2	−0.050	0.272	0.786	0.131	1.356	0.175	0.406	4.256	<0.001
S-5-ACC2	−0.092	0.490	0.624	0.015	0.134	0.893	0.401	4.020	<0.001
S-10-ACC2	−0.068	0.422	0.673	−0.007	0.061	0.951	0.399	4.185	<0.001
S-13-ACC2	−0.340	1.726	0.084	0.001	0.010	0.992	0.399	3.663	<0.001
S-17-ACC2	−0.215	1.097	0.273	0.037	0.369	0.712	0.407	3.892	<0.001

S, muscle strength; Arabic numerals, channel number; RT0, reaction time during 0-back; RT1, reaction time during 1-back; RT2, reaction time during 2-back; ACC0, accuracy during 0-back; ACC1, accuracy during 1-back; ACC2, accuracy during 2-back; Path A, muscle strength predicted the task-related hemodynamics; Path B, the task-related hemodynamics predicted working memory; Path C′, muscle strength predicted working memory.

**Table 5:** Mediation model test of HbO_2_.

Path mode	Path A			Path B			Path C′		
	Coefficient	*t*	*p*	Coefficient	*t*	*p*	Coefficient	*t*	*p*
S-15-RT0	−0.043	0.507	0.612	0.013	0.164	0.870	−0.462	8.026	<0.001
S-15-ACC0	−0.053	0.612	0.541	0.038	0.527	0.598	0.264	2.798	0.005
S-10-RT0	−0.108	1.178	0.239	−0.103	0.078	0.188	−0.474	7.520	<0.001
S-10-ACC0	−0.157	1.676	0.094	0.074	0.900	0.368	0.273	2.727	0.006
S-19-RT0	0.223	1.622	0.105	−0.158	1.945	0.052	−0.426	6.245	<0.001
S-19-ACC0	0.111	0.615	0.539	−0.024	0.241	0.809	0.260	2.574	0.01
S-23-RT0	−0.012	0.112	0.911	−0.076	0.700	0.484	−0.421	7.242	<0.001
S-23-ACC0	−0.011	0.103	0.918	−0.086	1.598	0.110	0.261	2.730	0.006
S-17-RT0	0.219	1.970	0.049	−0.091	0.969	0.332	−0.401	6.148	<0.001
S-17-ACC0	0.220	1.935	0.053	−0.017	0.167	0.868	0.266	2.628	0.009
S-2-RT1	0.134	1.223	0.221	−0.141	0.778	0.437	−0.441	4.914	<0.001
S-2-ACC1	0.159	1.484	0.138	0.190	1.801	0.072	0.419	5.762	<0.001
S-12-RT1	0.149	1.403	0.161	0.285	2.187	0.029	−0.506	7.741	<0.001
S-12-ACC1	0.144	1.409	0.159	−0.024	0.236	0.813	0.453	6.550	<0.001
S-22-ACC1	0.055	0.377	0.707	0.003	0.029	0.977	0.449	6.957	<0.001
S-19-RT1	0.223	1.657	0.098	−0.158	1.966	0.049	−0.426	6.389	<0.001
S-19-ACC1	0.072	0.749	0.454	0.077	0.627	0.531	0.444	6.751	<0.001
S-14-ACC2	0.009	0.071	0.944	−0.081	1.230	0.219	0.400	4.559	<0.001
S-15-ACC2	−0.005	0.054	0.957	−0.028	0.517	0.605	0.399	4.573	<0.001
S-20-ACC2	−0.013	0.113	0.91	−0.069	0.910	0.363	0.398	4.379	<0.001
S-16-ACC2	0.013	0.104	0.917	−0.026	0.267	0.790	0.400	4.393	<0.001
S-4-ACC2	−0.054	0.289	0.773	−0.053	0.687	0.492	0.396	4.072	<0.001
S-5-ACC2	0.087	0.517	0.605	−0.085	1.118	0.264	0.406	3.836	<0.001
S-10-ACC2	−0.046	0.280	0.780	−0.096	1.190	0.234	0.394	3.994	<0.001
S-17-ACC2	−0.068	0.403	0.687	−0.119	1.760	0.079	0.391	4.118	<0.001
S-17-ACC2	0.151	0.803	0.422	−0.025	0.319	0.750	0.402	4.031	<0.001
S-19-ACC2	0.077	0.642	0.521	0.076	0.981	0.327	0.393	4.411	<0.001

S, muscle strength; Arabic numerals, channel number; RT0, reaction time during 0-back; RT1, reaction time during 1-back; RT2, reaction time during 2-back; ACC0, accuracy during 0-back; ACC1, accuracy during 1-back; ACC2, accuracy during 2-back; Path A, muscle strength predicted the task-related hemodynamics; Path B, the task-related hemodynamics predicted working memory; Path C′, muscle strength predicted working memory.

The Smart PLS mediation effect test process was used to find that muscle strength had a significant impact on RT (i.e., 0-back, 1-back) and ACC (i.e., 0-back, 1-back, 2-back) when cortical hemodynamics were not considered, fulfilling the first step’s conditions. With the inclusion of cortical hemodynamics, muscle strength predicted the change of cortical hemodynamics (HbO_2_: channel 17, Hb: channel 4, 14, 22) during N-back task, indicating that path A was significant, but cortical hemodynamics did not predict WM performance, indicating that Path B was not significant, and the mediation analysis was discontinued. Similarly, cortical hemodynamics (HbO_2_: ch12, 19; Hb: ch9, 22) predicted WM performance, indicating that Path B was significant, but muscle strength did not predict cortical hemodynamics, indicating that Path A was not significant and stopping the mediation analysis.

Furthermore, the mediation effect test showed that the indirect effect was not significant, as the 95% confidence interval includes 0 (Tables 6, 7), indicating that there was no mediation effect in the relationship between muscle strength and WM.

**Table 6:** Hb mediation analysis.

Path model	Direct effect		Indirect effect		Total effect	
	c′	95%CI	a*b	95%CI	c	95%CI
S-8-RT0	−0.431	−0.562, -0.237	−0.03	−0.142, 0.029	−0.461	−0.560, −0.313
S-20-RT0	−0.412	−0.518, −0.265	−0.053	−0.205, 0.026	−0.465	−0.005, −0.556
S-22-RT0	−0.381	−0.483, −0.202	−0.039	−0.208, 0.007	−0.42	−0.518, −0.286
S-1-RT0	−0.428	−0.528, −0.286	0.009	−0.012, 0.051	−0.419	−0.517, −0.276
S-23-RT0	−0.420	−0.525, 0.284	0	−0.101, 0.022	−0.42	−0.516, −0.286
S-9-RT1	−0.479	−0.623, −0.315	−0.018	−0.096, 0.017	−0.497	−0.623, −0.323
S-9-ACC1	0.458	0.316, 0.527	0.011	−0.014, 0.052	0.469	0.332, 0.578
S-20-RT1	−0.417	−0.481, 0.026	−0.054	−0.176, 0.006	−0.471	−0.596, −0.297
S-20-ACC1	0.407	0.273, 0.525	0.040	−0.007, 0.123	0.447	0.304, 0.569
S-1-RT1	−0.463	−0.592, −0.195	−0.002	−0.074, 0.052	−0.465	−0.597, −0.182
S-1-ACC1	0.444	0.219, 0.561	0.004	−0.022, 0.077	0.448	0.243, 0.559
S-23-RT1	−0.467	−0.590, −0.301	0.003	−0.038, 0.043	−0.464	−0.592, −0.299
S-23-ACC1	0.453	0.306, 0.572	−0.003	−0.028, 0.018	0.450	0.302, 0.563
S-20-ACC2	0.385	0.152, 0.530	0.013	−0.023, 0.082	0.398	0.163, 0.536
S-22-ACC2	0.406	0.211, 0.569	−0.006	−0.075, 0.035	0.400	0.210, 0.543
S-5-ACC2	0.401	0.182, 0.568	−0.001	−0.062, 0.030	0.400	0.182, 0.546
S-10-ACC2	0.399	0.187, 0.561	0	−0.035, 0.054	0.399	0.184, 0.541
S-13-ACC2	0.399	0.146, 0.583	0	−0.076, 0.084	0.399	0.179, 0.536
S-17-ACC2	0.407	0.172, 0.577	−0.008	−0.086, 0.032	0.399	0.182, 0.538

S, muscle strength; Arabic numerals, channel number; RT0, reaction time during 0-back; RT1, reaction time during 1-back; RT2, reaction time during 2-back; ACC0, accuracy during 0-back; ACC1, accuracy during 1-back; ACC2, accuracy during 2-back; c′, direct effect; a*b, indirect effect; c, total effect.

**Table 7:** HbO_2_ mediation analysis.

Path model	Direct effect		Indirect effect		Total effect	
	c′	95%CI	a*b	95%CI	c	95%CI
S-20-RT0	−0.474	−0.578, −0.325	0.011	−0.006, 0.060	−0.463	−0.007, −0.561
S-19-RT0	−0.426	−0.539, −0.268	−0.035	−0.108, 0.002	−0.461	−0.559, −0.305
S-19-ACC0	0.260	−0.020, 0.420	−0.003	−0.005, 0.030	0.257	0.008, 0.407
S-23-RT0	−0.421	−0.520, −0.291	0.001	−0.020, 0.034	−0.420	−0.521, −0.285
S-17-RT0	−0.401	−0.509, −0.254	−0.020	−0.081, 0.013	−0.421	−0.519, −0.289
S-17-ACC0	0.266	−0.006, 0.424	−0.004	−0.059, 0.040	0.262	0.005, 0.410
S-2-RT1	−0.441	−0.591, −0.220	−0.019	−0.120, 0.024	−0.460	−0.593, −0.271
S-2-ACC1	0.419	0.251, 0.540	0.030	−0.003, 0.105	0.449	0.307, 0.557
S-12-RT1	−0.506	−0.617, −0.359	0.043	−0.013, 0.155	−0.463	−0.592, −0.302
S-12-ACC1	0.453	0.300, 0.574	−0.004	−0.047, 0.026	0.449	0.305, 0.560
S-22-ACC1	0.449	0.307, 0.559	0	−0.028, 0.032	0.449	0.305, 0.553
S-19-RT1	−0.426	−0.536, −0.263	−0.035	−0.115, 0.003	−0.461	−0.553, −0.310
S-19-ACC1	0.444	0.301, 0.555	0.003	−0.012, 0.065	0.447	0.307, 0.555
S-14-ACC2	0.400	0.177, 0.540	−0.001	−0.038, 0.023	0.399	0.175, 0.533
S-15-ACC2	0.399	0.174, 0.537	0	−0.013, 0.017	0.399	0.182, 0.537
S-20-ACC2	0.398	0.162, 0.534	0.001	−0.021, 0.039	0.399	0.165, 0.538
S-16-ACC2	0.400	0.165, 0.544	0	−0.035, 0.026	0.400	0.166, 0.543
S-4-ACC2	0.396	0.139, 0.545	0.003	−0.023, 0.058	0.399	0.152, 0.546
S-5-ACC2	0.406	0.087, 0.561	−0.007	−0.069, 0.016	0.399	0.085, 0.547
S-10-ACC2	0.394	0.090, 0.537	0.004	−0.039, 0.055	0.398	0.102, 0.538
S-13-ACC2	0.391	0.153, 0.539	0.008	−0.042, 0.068	0.399	0.161, 0.543
S-17-ACC2	0.402	0.155, 0.563	−0.004	−0.067, 0.024	0.398	0.146, 0.547
S-19-ACC2	0.393	0.181, 0.539	0.006	−0.008, 0.059	0.399	0.175, 0.539

S, muscle strength; Arabic numerals, channel number; RT0, reaction time during 0-back; RT1, reaction time during 1-back; RT2, reaction time during 2-back; ACC0, accuracy during 0-back; ACC1, accuracy during 1-back; ACC2, accuracy during 2-back; c′, direct effect; a*b, indirect effect; c, total effect.

## Discussion

This study investigated the correlation among muscle strength, working memory, and cortical hemodynamics during N-back task, and further explored whether cortical hemodynamics during N-back task mediated the relationship between muscle strength and WM performance. We observed that muscle strength (particularly grip strength) predicted WM of older adults in this cross-sectional study, which validated our hypothesis and expanded on previous research findings. Studies demonstrated that grip strength predicted executive function decline in patients with mild cognitive impairment (Herold et al., 2022). Other cross-sectional studies showed that grip strength and lower limb strength also predicted cognitive impairment (Veronese et al., 2016; Chou et al., 2019). Previous research revealed that grip strength was positively linked to cognitive functions such as WM, language fluency, and word recall (Ruan et al., 2020).

The reason why grip strength predicted working memory might be the control of muscles by the nervous system. Grip strength was influenced not only by muscle volume but also by the central nervous system, conversely, neurologic deterioration not only contributed to cognitive decline but might also be a factor in strength loss (Tian, 2020). This was consistent with the findings of the present study, where we found that greater muscle strength was associated with higher levels of activation in specific regions of the PFC and better WM performance. The greater the muscle strength, the stronger the activity of L-DLPFC at a low WM load (i.e., 0-back). At moderate, high WM load (i.e., 1-, 2-back), the greater the muscle strength, the more active areas (R-DLPFC, L-DLPFC, R-FPA, and L-FPA). Some studies suggested that the PFC played a crucial role in high grip strength performance, indicating that it may be the connection between grip strength and executive function (Dai et al., 2001). A systematic review found that resistance exercise improved brain function, particularly changes in the PFC, accompanied by improvements in executive function (Herold et al., 2019). Our findings further validated that a certain level of muscle strength was beneficial for brain health.

Furthermore, our finding that higher WM load was associated with fewer activation areas supported our hypothesis and was consistent with the compensation-related utilization of the neural circuit hypothesis, which suggested that older adults showed over-activations at a lower WM load, and under-activations at a higher WM load (Cappell et al., 2010). Previous research found that higher levels of oxyhemoglobin concentration in the PFC of older adults during cognitive tasks were associated with better cognitive performance, particularly in the DLPFC, which was closely linked to WM (Bierre et al., 2017). Additionally, studies showed that the level of PFC activation increased with increasing WM load in older adults, and then tended to stabilize or decrease (Mattay et al., 2006). Older adults exhibited greater DLPFC activation than younger adults during WM tasks (Turner and Spreng, 2012), and meta-analysis showed that when young people and older adults had the same cognitive performance, young people exhibited greater activity in L-VLPFC, while older people exhibited greater activity in L-DLPFC (Spreng et al., 2010). These findings suggested that older adults could compensate for cognitive performance by activating more task-related brain regions, supporting the assumption of a positive neurobiobehavioral relationship between cortical hemodynamics and cognitive performance.

Our study found cortical hemodynamics during N-back tasks did not mediate the relationship between muscle strength and WM performance. This was consistent with Herold’s work, which found that cortical hemodynamics during Sternberg task did not mediate the relationship between grip strength and WM in young adults (Herold et al., 2021). It suggested that the reason for the absence of a mediation effect could be related to the absence of a significant correlation between grip strength and WM, which was contrary to the results of the present study. The relationship between grip strength and higher cognitive functions has been demonstrated in both young (Kuwamizu, 2021) and older adults (Cui et al., 2021). The different results of the two studies may be due to different cognitive tests or differences in the participants. The reason for the absence of a mediation effect in this study was that although we observed significant correlations between task-related hemodynamics and muscle strength, and WM, these correlations were only in a few channels observed, and the correlation coefficient was very small (Table 8).

**Table 8:** Correlation between muscle strength and working memory performance in older adults.

	RT0	ACC0	RT1	ACC1	RT2	ACC2
MGS	−0.29***	0.29**	−0.45***	0.40***	−0.40**	0.33**
NGS	−0.24*	0.28*	−0.44***	0.33**	−0.37**	0.32**
30SUP	−0.35**	0.39***	−0.26*	0.42***	0.11	0.32**
30NSUP	−0.22*	0.2	0.09	0.25*	0.15	0.19

RT0,0-back reaction time; ACC0, 0-back accuracy; RT1, 1-back reaction time; ACC1, 1-back accuracy; RT2, 2-back reaction time; ACC2, 2-back accuracy; GS, grip strength; MGS, maximal grip strength; NGS, normalized grip strength; 30SUP, 30-s sit-up; 30NSUP, 30-s normalized sit-up; *p < 0.05; **p < 0.01; ***p < 0.001.

## Limitations

Although our findings are intriguing, this study has certain limitations that require further discussion. Firstly, the population under investigation comprised older adults residing in nursing homes. Given their age, their brain may have experienced some degree of atrophy. The characteristics of this research population restrict the generalizability of our results. Future research should seek to validate these findings in relatively younger older adults residing in the community. Secondly, while our sample size met the required standards, it was relatively small. Finally, heart disease, diabetes, hypertension, education level, and other factors may affect the results of the study, which should be considered as confounding factors in future research.

## Conclusion

The study showed a positive correlation between muscle strength, particularly grip strength, and WM in older adults. Higher levels of grip strength were associated with better WM performance. Furthermore, greater muscle strength was linked to increased activation in the prefrontal cortex during N-back task, indicating that muscle strength had a positive influence on brain health. It can be inferred that an increase in muscle strength was associated with prefrontal cortex activation, thereby promoting positive effects on brain health.

## Data availability statement

The original contributions presented in the study are included in the article/supplementary material, further inquiries can be directed to the corresponding author.

## Ethics statement

The studies involving humans were approved by Ethics Committee of Shanghai University of Sport. The studies were conducted in accordance with the local legislation and institutional requirements. The participants provided their written informed consent to participate in this study.

## Author contributions

XW contributed to the conception of the work. ZC contributed to the design and manuscript writing of the work. QW analyzed the data of the work. All authors contributed to the manuscript revisions, agreed with final approval of the version, and ensured the accuracy of research.
